# Prevalence and symptoms of Long Covid-19 in the workplace

**DOI:** 10.1093/occmed/kqae128

**Published:** 2025-01-11

**Authors:** H Mohd Yusoff, S Q Yew, A Mohammed Nawi, O Htwe, N Mohd Tohit, Z Mohamed, M A Muhamad Noordin, N Che Mohamed, F H Mohd

**Affiliations:** Department of Public Health Medicine, Faculty of Medicine, Universiti Kebangsaan Malaysia, Jalan Yaacob Latif, Bandar Tun Razak, Cheras, 56000 Kuala Lumpur, Malaysia; Department of Public Health Medicine, Faculty of Medicine, Universiti Kebangsaan Malaysia, Jalan Yaacob Latif, Bandar Tun Razak, Cheras, 56000 Kuala Lumpur, Malaysia; Department of Public Health Medicine, Faculty of Medicine, Universiti Kebangsaan Malaysia, Jalan Yaacob Latif, Bandar Tun Razak, Cheras, 56000 Kuala Lumpur, Malaysia; Department of Orthopaedics and Traumatology, Faculty of Medicine, Universiti Sultan Zainal Abidin, Gong Badak, 21300 Kuala Nerus, Terengganu, Malaysia; Department of Family Medicine, Faculty of Medicine, Universiti Kebangsaan Malaysia, Jalan Yaacob Latif, Bandar Tun Razak, Cheras, 56000 Kuala Lumpur, Malaysia; Negeri Sembilan State Health Department, Jalan Rasah, Negeri Sembilan, 70300 Seremban, Malaysia; Research Management Center, Research and Development Department, National Institute of Occupational Safety and Health, 43650 Bandar Baru Bangi, Selangor, Malaysia; Project Management, UKM Pakarunding, Level 3, Bangunan Wawasan, Universiti Kebangsaan Malaysia, 43600 Bangi, Selangor, Malaysia; Project Management, UKM Pakarunding, Level 3, Bangunan Wawasan, Universiti Kebangsaan Malaysia, 43600 Bangi, Selangor, Malaysia

## Abstract

**Background:**

The symptoms of Long coronavirus disease 2019 (Covid-19) are heterogeneous, creating uncertainty for employers regarding the diagnosis. The prevalence of Long Covid-19 in the workforce is also unknown. Furthermore, workers affected by Long Covid-19 encounter considerable difficulties in ensuring work safety and returning to their jobs due to this condition.

**Aims:**

This review is aimed to identify the prevalence of Long Covid-19 in the workplace and to determine the various symptoms of Long Covid-19 experienced by the workers.

**Methods:**

A meta-analysis was conducted to calculate the pooled estimates for the prevalence of Long Covid-19. Heterogeneity among the estimates was evaluated using the *I*² statistic.

**Results:**

The pooled prevalence of Long Covid-19 among workers across the 11 studies was 38% (95% CI 23–56). A total of 43 symptoms associated with Long Covid-19 were identified in the workplace, with the top five symptoms being dyspnoea at moderate activity (51%, 95% CI 39–62), mental symptoms (38%, 95% CI 6–87), dyspnoea at mild activity (35%, 95% CI 25–47), fatigue (26%, 95% CI 3–78) and effort intolerance (24%, 95% CI 15–35).

**Conclusions:**

The review indicates a significant burden of long-lasting symptoms within the workforce. The top five reported symptoms of Long Covid-19 were dyspnoea during mild and moderate activities, mental symptoms, fatigue and effort intolerance.

## Teaser

There were 43 symptoms associated with Long coronavirus disease (Covid-19) in the workplace. The prevalence of Long Covid-19 among workers was 38%. Common symptoms include dyspnoea, mental symptoms, fatigue and effort intolerance.

## Introduction

Long coronavirus disease 2019 (Covid-19) is a disease condition that has different definitions depending on the organization, country or experts involved. The World Health Organization (WHO) defines Long Covid-19 as the continuation or emergence of new symptoms three months after the initial severe acute respiratory syndrome coronavirus 2 (SARS-CoV-2) infection, with these symptoms lasting for at least 2 months without any other explanation [1]. On the other hand, the US Centre for Disease Control and Prevention provides a more specific definition, encompassing signs, symptoms and conditions that persist or develop after 4 weeks or more following the initial phase of Covid-19 infection, potentially leading to severe and life-threatening events even months or years later [2]. To further complicate matters, a diverse array of terminologies has been employed to describe this specific disease condition, including Long Covid-19 [3], chronic Covid-19 [4], post-Covid-19 [5], post-acute Covid-19 [6], persistent Covid-19 symptoms [7] and post-acute sequelae of SARS-CoV-2 infection [8].

Irrespective of the varied definitions and terminologies employed, this disease condition has resulted in a significant disease burden worldwide [9,10]. For instance, a recent meta-analysis consisting of 120 studies indicated a high global prevalence of Long Covid-19, in which almost 4 out of 10 (42%) individuals tested positive for Covid-19 infection were found to suffer from Long Covid-19 symptoms [11]. Of note, individuals affected by Long Covid-19 can present with a wide range of symptoms across multiple body systems [12,13]. In most cases, they may experience symptoms including fatigue, malaise and acute illness-induced fever [11]. Others may experience neurological symptoms such as difficulty in concentration, headache, altered taste and smell, cognitive impairment, memory deficits, as well as dizziness [11]. Also reported are cardiopulmonary symptoms such as dyspnoea, decreased effort tolerance and palpitations [12]. Alongside these physical symptoms, individuals affected by Long Covid-19 may also endure symptoms of post-traumatic stress disorder (PTSD), anxiety and depression, which can persist beyond a 6-month follow-up period [13]. The association between Long Covid-19 and these mental symptoms were established using self-administered questionnaires such as the Patient Health Questionnaire-9 (PHQ-9), Primary Care PTSD Screen (PC-PTSD), Trauma Screening Questionnaire and Generalized Anxiety Disorder Assessment (GAD-7) [14].

Owing to the extensive symptoms of Long Covid-19 as abovementioned, many employed individuals with Long Covid-19 face significant challenges when it comes to returning to work [15]. Coupled with the high workload and work-related stress in their original workplaces [16], as well as the slow pace of recovery from Covid-19, these group of individuals may also have increased rates of sick absenteeism [17]. These challenges can further prevent them from returning to work and may even lead to a sense of losing their profession [18]. Even if these individuals manage to return to work, the symptoms of Long Covid-19 can make it difficult for them to focus on their job tasks, leading to decreased work productivity [19]. Not to mention, Long Covid-19 symptoms can give rise to safety concerns for these employed individuals, particularly those who are exposed to multiple occupational hazards. As such, it comes as no surprise that the presence of Long Covid-19 symptoms is associated to a lower likelihood of working full-time (adjusted OR: 0.84, 95% CI 0.74–0.96) and a higher likelihood of unemployment (adjusted OR: 1.23, 95% CI 1.02–1.48) [20]. This loss of job and income, in turn, can further contribute to the deterioration of health status among the workers. All these findings collectively highlight the detrimental impacts of Long Covid-19 in the workplace.

Despite the established negative impacts of Long Covid-19 on the performance of workers across different industries, there remains a dearth of research exploring the prevalence of Long Covid-19 in the working population [21]. Additionally, even though Long Covid-19 prevalence has been extensively studied previously, it is important to note that these findings primarily reflect the general population and may not fully encompass the working population [22]. In short, it is still unclear what is the prevalence and symptoms of Long Covid-19 experienced by the workers. Recognizing these gaps in the literature, the objective of this review is to systematically appraise and synthesize the evidence on the prevalence of Long Covid-19 in the workplace. Secondly, this review is aimed to determine the various symptoms of Long Covid-19 experienced by the workers. Once the prevalence and symptoms of Long Covid-19 in the workplace are identified, it is hoped that this information will facilitate the employers, policymakers and various stakeholders to implement effective control measures in mitigating the adverse effects of Long Covid-19 in the workplace, while also providing comprehensive support to workers grappling with this disease condition.

## Methods

This systematic review and meta-analysis adhered to the Preferred Reporting Items for Systematic Reviews and Meta-Analyses (PRISMA) guidelines, ensuring comprehensive and transparent reporting. The research protocol was previously published in the International Prospective Register of Systematic Reviews, PROSPERO (ID CRD42024499679), further enhancing the study’s credibility and accountability.

Articles were included in the current review if they met the following inclusion criteria: (i) quantitative studies such as case-control, cohort, cross-sectional studies and randomized controlled trials, including preprints; (ii) studies that investigated the prevalence of Long Covid-19 among workers from all sectors; (iii) studies reported in the English language and (iv) studies published from 1 January 2020 onwards. Conversely, articles were excluded if they met any of the following criteria: (i) follow-up period of <12 weeks or unclear follow-up (based on the WHO’s Long Covid-19 definition); (ii) case series, case reports, qualitative studies and secondary analyses (e.g. systematic reviews) and (iii) grey literature.

A comprehensive search strategy was employed across three databases, namely PubMed, Web of Science and Scopus. PubMed, Web of Science and Scopus are renowned academic databases that collectively offer extensive coverage across various disciplines. Utilizing all three platforms ensures a more comprehensive and inclusive search strategy, allowing researchers to access a diverse range of scholarly articles, journals and conference proceedings relevant to the systematic literature review. To ensure completeness, the search in these databases was complemented by a manual examination of references cited in relevant systematic reviews.

For the systematic search, a robust Boolean search strategy was implemented, incorporating a blend of Medical Subject Headings and appropriate text words. The search terms were crafted in collaboration with the research team and relevant stakeholders to encompass all relevant aspects of the subject matter. A table outlining the search terms and search strategies for the three databases was presented in the Supplementary material (available as Supplementary data at *Occupational Medicine* online).

All studies identified through the search strategy underwent a rigorous assessment for eligibility. Initially, title and abstract screening were conducted independently by two researchers (S.Q.Y. and H.M.Y.). Subsequently, a full-text screening was performed to further evaluate the suitability of the studies. In cases where multiple publications originated from the same study, only the most recent publication was included in the analysis, ensuring avoidance of duplication.

A structured data extraction form in Microsoft Office Excel was utilized to extract relevant information from each eligible study. The extracted data encompassed details such as author names, countries, study designs, industrial sectors, details of study population, severity of Covid-19 of study population, sample sizes, participant characteristics (including age and gender), diagnostic method, minimum duration of follow-up and prevalence of Long Covid-19 symptoms. For studies that presented data on the prevalence of individual symptoms without providing an overall prevalence of Long Covid-19, we determined the overall prevalence by referring to the symptom with the highest prevalence. Two researchers (S.Q.Y. and H.M.Y.) independently performed the data extraction, and any discrepancies were resolved through discussions involving a third researcher (A.M.N.).

The primary outcome of our analysis was the prevalence of Long Covid-19 in the workplace. Subsequently, we conducted further analyses to examine how the prevalence of Long Covid-19 in the workplace varied across different factors, including study designs, industrial sectors, follow-up duration, age of workers, gender of workers, diagnostic method, methods of symptom assessment, presence of a control group or the definition of Long Covid-19.

To evaluate the quality of the included studies, the Joanna Briggs Institute (JBI) Critical Appraisal Checklist for Studies Reporting Prevalence Data was employed [23]. The selection of the JBI Critical Appraisal Checklist is grounded in its evidence-based practice focus, versatility across study designs, inclusion of qualitative research criteria, user-friendly format, alignment with systematic review standards, continuous updates, international recognition and comprehensive evaluation of methodological rigor. The checklist consists of nine items, each with four possibilities for assessment. A qualitative assessment was conducted on each of the nine items to determine the overall quality rating of each study, categorized as good, moderate or low. Two researchers (S.Q.Y. and H.M.Y.) independently assessed the quality, and any disagreements were resolved through discussions involving a third researcher (A.M.N.). It is important to note that the studies were not excluded based on their quality. However, a sensitivity analysis was conducted to evaluate the influence of study quality (i.e. risk of bias) on the study outcome, whereby studies with low quality were excluded and the estimated pooled prevalence of Long Covid-19 was recalculated.

A meta-analysis was conducted to calculate the pooled estimates for the prevalence of Long Covid-19 in the workplace with a 95% CI as an estimated effect across studies. Heterogeneity among the estimates was evaluated using the *I*² statistic. Random-effects or fixed-effects models were employed, depending on the level of heterogeneity (*I*²). Fixed-effects models were utilized when studies exhibited low heterogeneity (*I*² ≤ 50%), while random-effects models were employed for studies with significant heterogeneity (*I*² ≥ 50%). Subgroup analyses were conducted to examine the pooled prevalence of Long Covid-19 in relation to study designs, industrial sectors, follow-up duration, age of workers, gender of workers, diagnostic method, methods of symptom assessment, presence of a control group or the definition of Long Covid-19. Publication bias was assessed by visually inspecting funnel plots and using the Egger bias test, with a *P*-value < 0.05 indicative of possible publication bias. All statistical analyses were performed using Comprehensive Meta-Analysis version 2.

## Results

**Figure 1.**
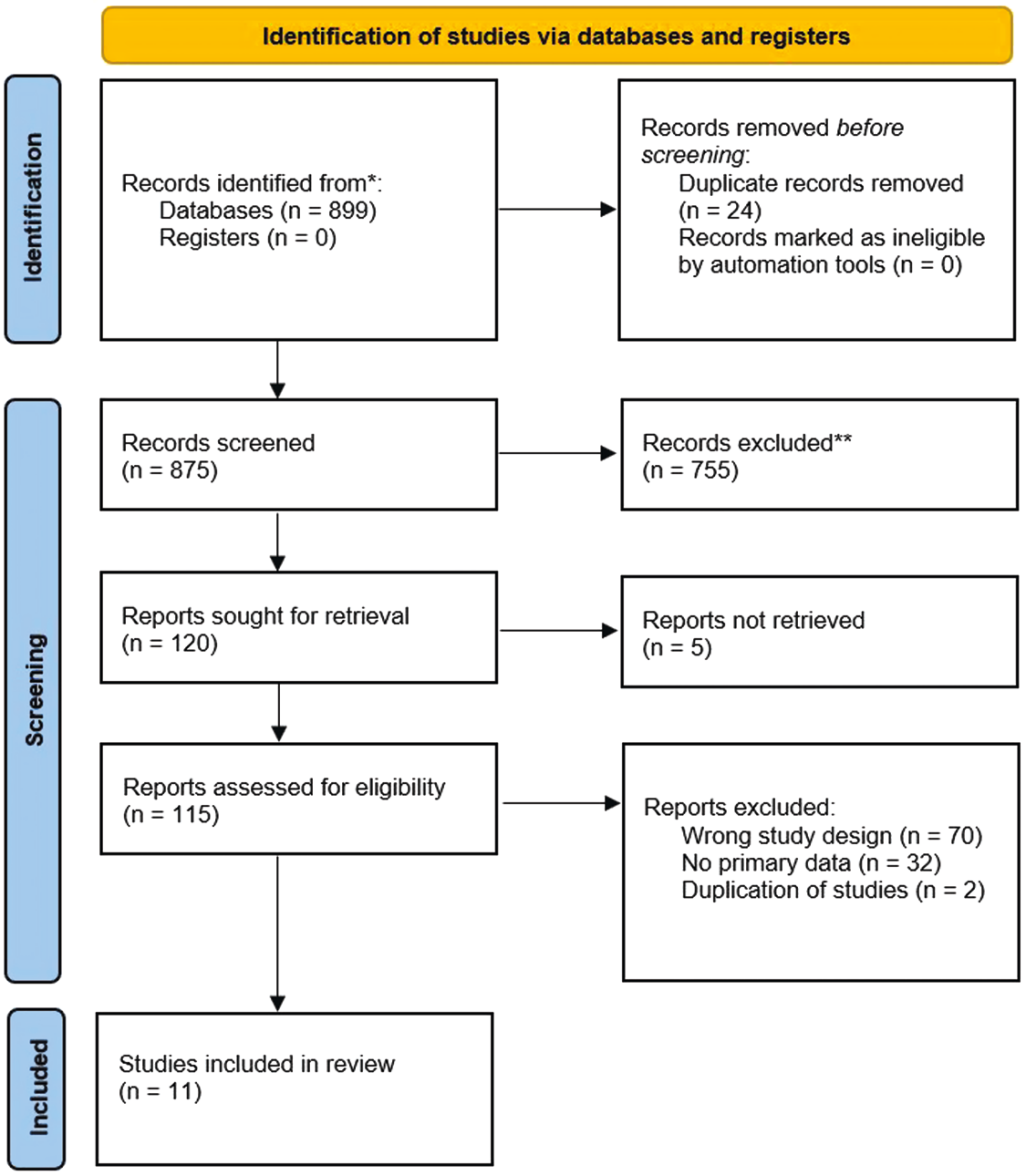
PRISMA flow diagram.

A total of 899 titles were identified through database searches for this review but only 11 were deemed suitable for inclusion (see Figure 1). As for the quality assessment, six of the included studies were classified as good and five as moderate. This implies that all 11 studies considered in this review carry a low risk of bias, as indicated in Table 1.

**Table 1.** Quality assessment by the JBI critical appraisal tool for prevalence studies

	1. Was the sample frame appropriate to address the target population?	2. Were study participants sampled in an appropriate way?	3. Was the sample size adequate?	4. Were the study subjects and the setting described in detail?	5. Was the data analysis conducted with sufficient coverage of the identified sample?	6. Were valid methods used for the identification of the condition?	7. Was the condition measured in a standard, reliable way for all participants?	8. Was there appropriate statistical analysis?	9. Was the response rate adequate, and if not, was the low response rate managed appropriately?	Overall
Bernas	No	NA	Yes	Unclear	Yes	Yes	Yes	Yes	No	Moderate
Gaber	Yes	Unclear	Yes	No	Yes	Yes	Yes	No	NA	Moderate
Ladlow	Yes	Unclear	No	Yes	Yes	Yes	Yes	Yes	NA	Moderate
Lemhofer	Yes	Yes	Yes	Yes	Yes	Yes	Yes	Yes	NA	Good
Martinez	Yes	Yes	Yes	Yes	Yes	Unclear	Unclear	Yes	Yes	Good
Perisse	Yes	Yes	Yes	Yes	Yes	Yes	Yes	Yes	Yes	Good
Pilmis	Yes	Unclear	No	Yes	Yes	Yes	Yes	Yes	Yes	Good
Selvaskandan	Yes	Yes	Yes	No	Yes	Unclear	Yes	Yes	NA	Moderate
Shukla	Yes	Unclear	Yes	Yes	Yes	Yes	Yes	Yes	Yes	Good
Stufano	Yes	Unclear	No	Yes	Yes	Yes	Yes	Yes	NA	Moderate
Tempany	Yes	No	Yes	Yes	Yes	Yes	Yes	Yes	No	Good

NA = Not applicable.

The studies encompassed a participant range of 71 to 12 609, totalling 14 979 participants in this review. The time elapsed from Covid-19 infection to participant recruitment varied from 12 to 24 weeks. The age of the cohorts spanned from 31.5 to 50.6 years, with five studies focussing on participants under 40 years old [24–28]. Geographically, the research was primarily conducted in Europe (*n* = 10) [24–27,29–34] and Asia (*n* = 1) [28]. The study designs included eight cross-sectional studies [24,26,28–30,32–34] and three prospective cohort studies [25,27,31].

The participants were drawn from diverse sectors, including healthcare (*n *= 6) [26,28,29,31,32,34], general industry (*n* = 2) [24,30], military (*n* = 2) [25,27] and education (*n* = 1) [33]. Notably, eight studies had a higher proportion of female participants [24,26,28–32,34]. Reverse transcription polymerase chain reaction was the chosen method for diagnosing Covid-19 infection in seven studies [24,27–29,31,33,34], while one study used the enzyme-linked immunosorbent assay [25]. Six studies categorized participants based on hospitalization status [24–26,28–30], while others classified them according to disease severity [27,33,34] or had undefined categorization [31,32]. Table 1 (available as Supplementary data at *Occupational Medicine* Online) summarizes the characteristics and primary outcomes of the 11 studies included in this review.

**Figure 2.**
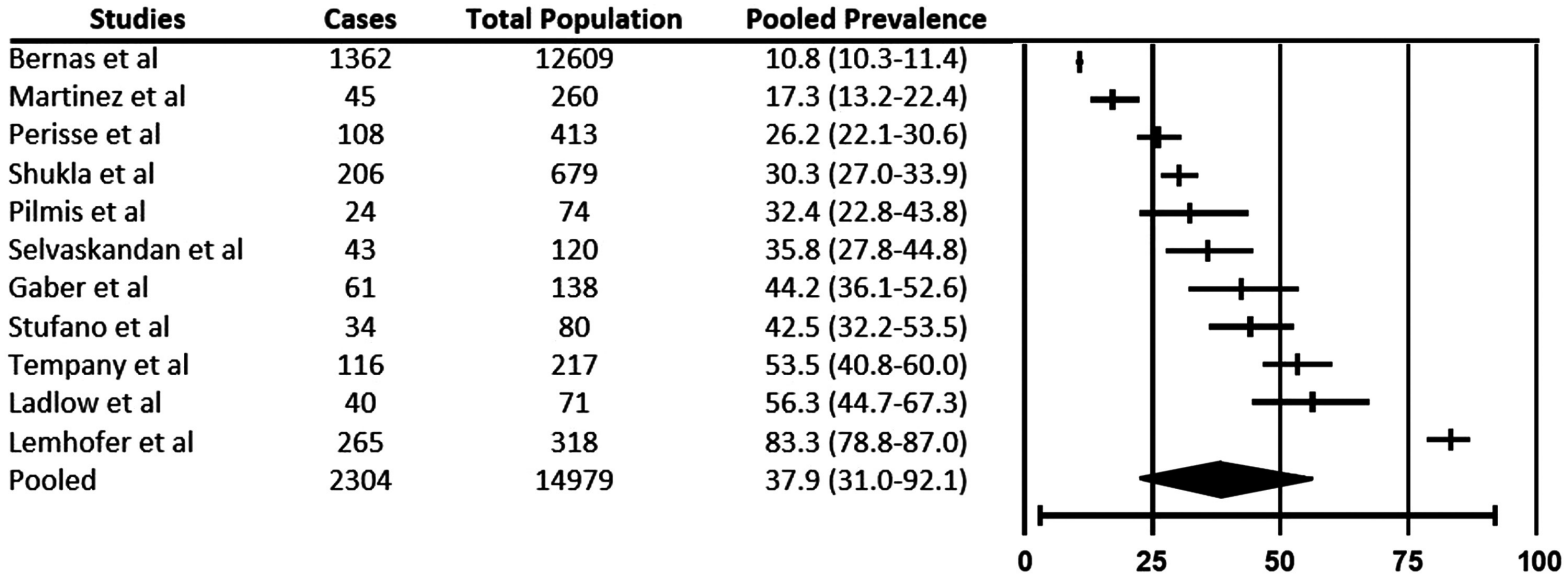
Pooled prevalence of Long Covid-19 among workers.

In this review, the prevalence of Long Covid-19 among workers varied from 10% to 54%, with a pooled prevalence of 38% (95% CI 22.5–56.2) as illustrated in Figure 2. In other words, the overall estimated prevalence across the 11 studies was 38%. Due to substantial heterogeneity among the studies (*I*^2^ = 99%), subgroup analyses were conducted. These analyses revealed that the prevalence of Long Covid-19 did not significantly differ based on industrial sectors, follow-up duration, age of workers, gender of workers, diagnostic methods, methods of symptom assessment, presence of a control group, provision of Long Covid-19 definition and study designs. The result of the subgroup analysis was provided as Supplementary material (available as Supplementary data at *Occupational Medicine* online).

Of note, we identified 43 symptoms associated with Long Covid-19 in the workplace, as detailed in Table 2. Among the participants, the five most commonly reported symptoms were dyspnoea at moderate activity (51%, 95% CI 39–62, *n* = 1), mental symptoms (38%, 95% CI 6–87, *n* = 2), dyspnoea at mild activity (35%, 95% CI 25–47, *n* = 1), fatigue (26%, 95% CI 3–78, *n* = 10) and effort intolerance (24%, 95% CI 15–35, *n* = 1). The asymmetry of the funnel plot and the *P*-value of <0.05 in Egger’s test suggest the presence of publication bias in this review.

**Table 2.** Long Covid-19 symptoms in the workplace

Body systems	Symptoms	Events	Total sample size	No. of studies	Pooled prevalence (%)	95% CI
General	Fatigue	4134	14 843	10	26	3–78
	Generalized weakness	32	331	2	11	6–20
	Fever	48	1090	4	5	1–22
	Reduced appetite	151	13 548	3	1	0–1
	Sleep disturbance	1844	14 705	8	22	10–34
Psychiatric	Mental disorders	247	533	2	38	6–87
	Anxiety	1250	13 701	3	3	1–10
	Depression	866	13 288	2	3	1–15
	Stress	7	679	1	1	1–2
	Mood disorders	51	258	2	15	2–56
Musculoskeletal	Arthralgia	972	13 619	4	7	1–51
	Myalgia	723	13 439	4	11	5–23
	Musculoskeletal pain	9	260	1	4	2–7
Nervous	Poor concentration	1255	14 105	6	10	6–16
	Confusion	6	71	1	9	4–18
	Dizziness	19	331	2	7	2–21
	Headache	796	14 112	6	7	5–9
	Syncope	1	71	1	1	0–9
	Poor attention	14	71	1	20	12–31
	Poor memory	839	13 359	3	7	2–23
	Tingling in extremities	7	679	1	1	1–2
	Cognitive impairment	37	217	1	17	13–23
Dermatological	Skin rash	8	759	2	1	1–2
	Pruritus	18	413	1	4	3–7
	Hair loss	8	260	1	3	2–6
Cardiovascular	Palpitations	396	13 619	4	4	1–11
	Chest pain	262	14 032	5	4	1–10
	Effort intolerance	17	71	1	24	15–35
Respiratory	Cough	445	14 112	6	5	3–10
	Dyspnoea	468	13 507	6	10	3–28
	Dyspnoea (at rest)	12	71	1	17	10–27
	Dyspnoea (at mild activity)	25	71	1	35	25–47
	Dyspnoea (at moderate activity)	36	71	1	51	39–62
Gastrointestinal	Digestive problems	14	673	2	2	1-6
	Diarrhoea	192	13 439	4	2	1–7
	Sore throat	135	13 439	4	3	1–7
	Abdominal pain	184	13 359	3	2	1–4
	Dysphagia	2	71	1	3	1–11
	Nausea/vomiting	57	13 288	2	1	0–2
Ear, nose and throat	Ear pain	3	679	1	0	0–1
	Vertigo	265	12 609	1	2	1–2.4
	Tinnitus	2	679	1	0	0–1
	Anosmia/ageusia	1125	14 232	6	10	6–16
Others	**–**	40	477	2	6	1–41

## Discussion

This systematic review aimed to evaluate the prevalence and symptoms of Long Covid-19 in the workplace, aligning with the original PROSPERO protocol. The findings reveal a significant prevalence of Long Covid-19 among workers, estimated at 38%. Intriguingly, this pooled prevalence is lower than those reported in meta-analyses conducted in the general population (ranging from 42% to 57% for Long Covid-19) [11–13,35]. This disparity may be attributed to the healthy worker effect, where individuals in better health are more likely to be employed, potentially resulting in a lower prevalence of Long Covid-19 among workers [36]. Conversely, those with poor health conditions, including those suffering from Long Covid-19 symptoms, may face challenges in securing and maintaining employment. Additionally, the employed population typically enjoys enhanced access to healthcare services, encompassing preventive care and early treatment, contributing to improved health outcomes compared to the unemployed. Another plausible explanation is that the participants in our review, namely workers, were potentially younger than the general population, potentially resulting in a lower prevalence of Long Covid-19 [11–13,35]. A third possible rationale could be that our review exclusively enrolled participants who experienced symptoms persisting beyond 12 weeks from the onset of acute Covid-19 infection, in contrast to prior analyses that encompassed participants displaying symptoms within the initial 12-week period [12].

Moreover, our review revealed that Long Covid-19 imposes a substantial burden of functional impairment, symptoms and pathology affecting various organ systems. This can be attributed to the multifaceted and potentially interconnected pathophysiological mechanisms of Long Covid-19, encompassing persisting viral reservoirs, immune dysfunction, micro-clotting, organ damage and psychological impacts [37,38]. The top five prevalent symptoms include dyspnoea at moderate activities (51%), mental symptoms (38%), dyspnoea at mild activities (35%), fatigue (26%) and effort intolerance (24%). Despite the lower overall prevalence of Long Covid-19 as compared to the previous meta-analyses, the prevalences of these top five symptoms were higher compared to the previous analyses. For instance, the prevalence of dyspnoea at moderate activities in our review (51%) exceeded that reported in earlier analyses (20–34%) [11–13,35]. Similarly, our analysis showed a higher prevalence of mental symptoms (38%) among our participants compared to the previous studies (8–19%) [11–13,35]. A similar trend was observed in symptoms such as dyspnoea at mild activities (35%), fatigue (26%) and effort intolerance (24%), where previous analyses reported a prevalence of 15–24% [11–13,35], 22–31% [11–13,35] and 17% [13], respectively. This discrepancy can be elucidated by the fact that a majority of our participants were drawn from stressful working conditions, such as healthcare and military sectors. These populations are potentially exposed to higher workloads and occupational stress [39,40].

Furthermore, our findings lend support to the notion that Covid-19 may result in the persistence of symptoms even after the resolution of acute infection, a phenomenon previously observed with SARS-CoV-1 and Middle East respiratory syndrome (MERS)-CoV. The SARS-CoV-1 pandemic initially emerged in Guangdong Province, China in 2003 and subsequently extended its reach to 29 countries [41]. A prospective cohort study conducted on individuals who survived SARS-CoV-1 infection revealed notable declines in diffusing capacity for carbon monoxide, exercise capacity and overall health status at 24 months post-infection [42], with healthcare workers experienced a more pronounced negative impact. Regarding psychological effects, workers who contracted SARS-CoV-1 reported significantly elevated levels of burnout, psychological distress and PTSD between 13 and 26 months after infection [43]. Remarkably, even 18 years later, fatigue remained the predominant symptom among the workers, and their respiratory function was notably lower compared to controls. Nonetheless, it is worth noting that emotional and mental health fully recovered at the 18-year follow-up [44]. In the case of MERS-CoV, a pandemic originating in Jeddah, Saudi Arabia in 2012 [45], a year-long follow-up revealed that 48% of survivors continued to experience fatigue symptoms, which decreased to 33% at 18 months of follow-up [46]. While 88% of individuals who survived MERS-CoV returned to work, the study did not specify the proportion engaged in part- or full-time employment [47]. Regarding mental health, 27% of survivors reported depression and 42% had PTSD at 12 months post MERS-CoV infection [46]. Another study conducted in South Korea demonstrated that 57% of nurses who treated MERS-CoV patients suffered from PTSD [48]. This evidence suggests that similar to SARS-CoV-1 and MERS-CoV, we should anticipate the long-term consequences of Covid-19, which will negatively impact the work performance of the workers. More concerning is the possibility that Covid-19 could lead to more extensive long-term consequences, given that SARS-CoV-2 has higher transmissibility and a greater capacity to mutate to different variants compared to SARS-CoV-1 and MERS-CoV [21].

The review has several strengths. First of all, we included an extensive electronic search for pertinent studies and a thorough evaluation of risk of bias, data extraction and verification, all conducted independently by two authors. Additionally, we employed the JBI critical appraisal tool for prevalence studies, ensuring a comprehensive assessment of study quality. The application of this tool indicated that all studies included in our review achieved moderate and/or high quality.

Nevertheless, it is crucial to acknowledge certain limitations in this review. Firstly, there was a notable heterogeneity between studies and a considerable range in prevalence. While earlier analysis pointed to the high heterogeneity resulting from the diverse array of self-report screening tools used to collect Long Covid-19 symptoms [11], our subgroup analysis did not reveal a similar pattern. Secondly, some prior analyses suggested that the introduction of control groups in certain studies might contribute to heterogeneity [11], yet this aspect is not apparent in our current analysis. Another noteworthy limitation that warrants attention is our use of the most commonly reported symptom estimate for individual studies, without combining multiple individual symptoms into an overall estimate of Long Covid-19 prevalence. Given the variability in the symptom with the highest prevalence across studies, direct comparisons may be challenging. Furthermore, similar to observations in other analyses, the lack of a unified consensus on the definition of Long Covid-19 poses a challenge, particularly concerning the varying time components for persistent symptoms following the infection onset [12]. It is essential to acknowledge that certain Long Covid-19 symptoms might be absent in some studies due to non-identification or non-inclusion in patient questionnaires [13]. In addition, the presence of publication bias in our findings is likely attributed to the decision to screen papers exclusively in English and the utilization of only three databases for screening [49]. By excluding non-English studies, the review may suffer from language bias, which can lead to an overrepresentation of studies from English-speaking countries. This can skew the findings if the results differ systematically by languages and geographical locations. Meanwhile, retrieving articles from a limited number of databases may miss important studies that are indexed in other databases.

Comprehensive management of Long Covid-19 symptoms, which affect multiple organs, necessitates the involvement of a diverse clinical team rather than relying on a specific specialty. Employers should be capable of identifying Long Covid-19 symptoms in their workers and offer prompt assistance, including potential adjustments to job activities. Insurers must address Long Covid-19, providing financial and vocational support to affected workers. If termination occurs due to Long Covid-19 symptoms, affected individuals should receive compensation.

This systematic review offers the latest and most extensive insights into the prevalence and symptoms of Long Covid-19 in the workplace, especially in the healthcare setting. The findings highlight that a considerable number of workers continue to grapple with a variety of persistent symptoms, with dyspnoea during mild and moderate activities, mental symptoms, fatigue and effort intolerance emerging as particularly prevalent. It is important to exercise caution in interpreting our prevalence estimates and the symptoms of Long Covid-19, given the substantial heterogeneity among studies resulting from diverse study designs.

**Key learning points**

**What is already known about this subject:**

- Workers with Long Covid-19 often report decreased work productivity due to persistent symptoms.
- There is a higher rate of sickness absenteeism as workers manage ongoing health issues.
- Employers may suffer a rise in healthcare costs due to the long-term medical needs of workers affected by Long Covid-19.

**What this study adds:**

- There were 43 symptoms associated with Long Covid-19 in the workplace.
- The prevalence of Long Covid-19 among workers was 38%.
- Common symptoms include dyspnoea, mental disorders, fatigue and effort intolerance.

**What impact this may have on practice or policy:**

- Awareness of Long Covid-19 symptoms enables employers to provide accommodations such as flexible hours or remote work.
- Addressing mental health impacts encourages better support and access to resources for affected employees.
- Early identification and support can reduce absenteeism by helping workers manage symptoms effectively.

## Supplementary Material

kqae128_suppl_Supplementary_Material

kqae128_suppl_Supplementary_Table_S2

kqae128_suppl_Supplementary_Table_S1

## Data Availability

Data are not publicly available. However, it will be made available upon request.
