# Supplementary material for: Prevalence and symptoms of Long Covid-19 in the workplace
Source: Occup Med (Lond). 2025 Jan 11;75(1):33–41. doi: 10.1093/occmed/kqae128 (PMC11973416; doi:10.1093/occmed/kqae128)
Supplement: kqae128_suppl_Supplementary_Material [file kqae128_suppl_supplementary_material.docx]

**Supplementary File: Search Strategies**

**PubMed**

(“Prevalence” OR “incidence” OR “frequency”) AND (“long covid*” OR “long-haul covid*” OR “chronic covid*” OR “post-Covid*” OR “post-acute Covid*” OR “persistent Covid*” OR “post-acute sequelae of SARS-CoV-2 infection”) AND (“Work*” OR “occupation*” OR “employ*” OR “job*”)

**Scopus**

TITLE-ABS-KEY(("Prevalence" OR "incidence" OR "frequency") AND ("long covid*" OR "long-haul covid*" OR "chronic covid*" OR "post-Covid*" OR "post-acute Covid*" OR "persistent Covid*" OR "post-acute sequelae of SARS-CoV-2 infection") AND ("Work*" OR "occupation*" OR "employ*" OR "job*"))

**Web of Science**

TS=("Prevalence" OR "incidence" OR "frequency") AND TS=("long covid*" OR "long-haul covid*" OR "chronic covid*" OR "post-Covid*" OR "post-acute Covid*" OR "persistent Covid*" OR "post-acute sequelae of SARS-CoV-2 infection") AND TS=("Work*" OR "occupation*" OR "employ*" OR "job*")
