# Supplementary material for: Prevalence and symptoms of Long Covid-19 in the workplace
Source: Occup Med (Lond). 2025 Jan 11;75(1):33–41. doi: 10.1093/occmed/kqae128 (PMC11973416; doi:10.1093/occmed/kqae128)
Supplement: kqae128_suppl_Supplementary_Table_S2 [file kqae128_suppl_supplementary_table_s2.docx]

**Table 2: Summary of the characteristics and the primary outcome of the 15 included studies**

| **Author (Year)** | **Countries** | **Study Designs** | **Industrial Sectors** | **Details of Study Population** | **Severity of Covid-19 of Study Population*** | **Sample Sizes** | **Age (years)** | **Gender (%)** | **Diagnostic Method**** | **Minimum Duration of Follow-Up** | **Prevalence (%)** |
| --- | --- | --- | --- | --- | --- | --- | --- | --- | --- | --- | --- |
| Bernas et al. (2023) [23] | Germany | Cross-sectional study | General | Potential stem cell donors registered with DKMS Germany | Participants were categorised into asymptomatic, moderate, severe, and hospitalisation. | 12,609 | Median: 38.0 | Male: 3318 (26.3) Female: 9291 (73.7) | RT-PCR | 12 | 28 |
| Gaber et al (2021) [28] | United Kingom | Cross-sectional study | Healthcare | Healthcare providers at the Wrightington, Wigan and Leigh NHS Teaching Trust | Participants were categorised into hospitalised (n = 3) and non-hospitalised (n = 135). | 138 | Not reported | Male: 23 (16.7) Female: 115 (83.3) | RT-PCR or SARS-CoV-2 Antibodies | 12 | 44 |
| Ladlow et al (2023) [24] | United Kingom | Prospective cohort study | Military | Personnel recruited from local military units. | Participants were categorised into hospitalised- symptomatic (n = 25), hospitalised-recovered (n = 6), community-symptomatic (n = 28), and community-recovered (n = 12). | 71 | Mean: 39 .0± 11.0 (non-hospitalised), 42.0 ± 8.0 (hospitalised) | Male: 61 (85.9) Female: 10 (14.1) | Spike and Anti-Nucleocapsid SARS-CoV2 Test | 20 | 56 |
| Lemhöfer et al (2023) [29] | Germany | Cross-sectional study | General | All patients presented to the Post-Covid outpatient clinic at Jena University Hospital | Participants were categorised into hospitalised (n = 75) and non-hospitalised (n = 243). | 318 | Mean: 46.9 ± 10.9 | Male: 99 (31.1) Female: 219 (68.9) | Not defined | 12 | 83 |
| Martinez et al (2021) [25] | Switzerland | Cross-sectional study | Healthcare | Employees at the University Hospital Basel | Participants were categorised into hospitalised (n = 3), hospitalised to ICU (n = 0), and non-hospitalised (n = 257). | 260 | Median: 37 (IQR 28–49) | Male: 64 (24.6) Female: 196 (75.4) | Not defined | 12 | 17 |
| Perisse et al (2023) [26] | France | Prospective cohort study | Military | Sailors onboard of the AC-CDG aircraft carrier. | Participants were categorised into asymptomatic, mild, moderate or severe. | 413 | Mean: 31.7 | Male: 368 (89.1) Female: 45 (10.9) | RT-PCR | 24 | 54 |
| Pilmis et al (2022) [30] | France | Prospective cohort study | Healthcare | Healthcare workers at the Saint-Joseph Hospital (physicians, nurses, hospital assistants, and hospital administrative staff). | Not defined | 74 | Median: 47.0 (IQR 33.2–54.2) | Male: 13 (17.6) Female: 61 (82.4) | RT-PCR | 12 | 32 |
| Selvaskandan et al (2022) [31] | United Kingom | Cross-sectional study | Healthcare | Members of the UK Kidney Association (doctors, nurses, technicians, pharmacists, psychologists and social workers). | Not defined | 120 | Not reported | Male: 38 (31.7) Female: 82 (68.3) | Not defined | 12 | 36 |
| Shukla et al (2022) [27] | India | Cross-sectional study | Healthcare | Healthcare workers at eight tertiary care hospitals. | Participants were categorised into hospitalised (n = 310), and non-hospitalised (n = 369). | 679 | Mean: 31.5 ± 9.5 | Male: 334 (49.2) Female: 345 (50.8) | RT-PCR | 12 | 30 |
| Stufano et al (2023) [32] | Italy | Cross-sectional study | Education | Employees of an Italian university (full professor, associate professor, senior researcher, and tenure-tracked researcher, technical clerk, or administrative employee). | Participants were categoried into asymptomatic, mild, moderate, severe. | 80 | Mean: 50.6 ± 9.3 | Male: 56 (70.0) Female: 24 (30.0) | RT-PCR | 16 | 43 |
| Tempany et al (2021) [33] | Ireland | Cross-sectional study | Healthcare | Employees of the Tallaght University Hospital. | Participants were categorised into known Covid-19 infection (n = 139) and assumed Covid-19 infection (n = 78). | 217 | Not reported | Male: 43 (19.8) Female: 174 (80.2) | RT-PCR | 12 | 54 |

*Disease severity was either classified according to the status of symptoms (i.e., asymptomatic, mild, moderate or severe) or status of hospitalisation (e.g., hospitalised or not hospitalised) or a combination of both.

**The diagnostic methods utilised in the included studies were either reverse transcription polymerase chain reaction (RT-PCR), SARS-CoV-2 antibodies, Spike and Anti-Nucleocapsid SARS-CoV2 test or a combination of the above.
