# Supplementary material for: Prevalence and symptoms of Long Covid-19 in the workplace
Source: Occup Med (Lond). 2025 Jan 11;75(1):33–41. doi: 10.1093/occmed/kqae128 (PMC11973416; doi:10.1093/occmed/kqae128)
Supplement: kqae128_suppl_Supplementary_Table_S1 [file kqae128_suppl_supplementary_table_s1.docx]

**Subgroup analysis**

| **Subgroups** | **Number of Studies** | **Pooled Prevalence** | **95% CI** | **P Value (Between Subgroups)** |
| --- | --- | --- | --- | --- |
| **Industrial Sectors** |  |  |  |  |
| Healthcare | 6 | 34.7 | 25.0-45.9 | 0.795 |
| General Industry | 2 | 43.7 | 2.0-96.7 |  |
| Military | 2 | 39.9 | 15.8-70.2 |  |
| Education | 1 | 42.5 | 32.2-53.5 |  |
|  |  |  |  |  |
| **Follow-up Duration** |  |  |  |  |
| 12 Weeks | 8 | 36.8 | 18.7-59.7 | 0.801 |
| Less than 12 Weeks | 3 | 40.6 | 23.4-60.3 |  |
|  |  |  |  |  |
| **Age of Workers** |  |  |  |  |
| Less than 40 years old | 5 | 25.5 | 13.7-42.3 | 0.222 |
| 40-50 years old | 2 | 61.0 | 13.6-93.9 |  |
| More than 50 years old | 1 | 42.5 | 32.2-53.5 |  |
|  |  |  |  |  |
| **Genders of Workers** |  |  |  |  |
| Predominantly Male | 3 | 40.6 | 23.4-60.3 | 0.801 |
| Predominantly Female | 8 | 36.8 | 18.7-59.7 |  |
|  |  |  |  |  |
| **Diagnostic Methods** |  |  |  |  |
| PCR | 7 | 32.4 | 18.0-51.0 | 0.100 |
| ELISA | 1 | 56.3 | 44.7-67.3 |  |
|  |  |  |  |  |
| **Methods of Symptom Assessment** |  |  |  |  |
| Self-Report | 9 | 38.3 | 19.7-61.1 | 0.075 |
| Interview | 1 | 30.3 | 27.0-33.9 |  |
| Mixed (Self-Report + Interview) | 1 | 42.5 | 32.2-53.5 |  |
|  |  |  |  |  |
| **Presence of Control Group** |  |  |  |  |
| Yes | 3 | 32.5 | 8.3-71.9 | 0.719 |
| No | 8 | 40.1 | 5.9-87.8 |  |
|  |  |  |  |  |
| **Provision of Long Covid-19 Definition** |  |  |  |  |
| Yes | 6 | 46.0 | 13.4-82.5 | 0.498 |
| No | 5 | 31.7 | 23.2-41.6 |  |
|  |  |  |  |  |
| **Study Designs** |  |  |  |  |
| Cross-Sectional | 8 | 38.1 | 19.4-61.1 | 0.954 |
| Prospective Cohort | 3 | 37.2 | 21.5-56.2 |  |
